# Optical Nanoscopy of Cytokine-Induced Structural Alterations of the Endoplasmic Reticulum and Golgi Apparatus in Insulin-Secreting Cells

**DOI:** 10.3390/ijms251910391

**Published:** 2024-09-27

**Authors:** Licia Anna Pugliese, Valentina De Lorenzi, Marta Tesi, Piero Marchetti, Francesco Cardarelli

**Affiliations:** 1 NEST Laboratory—Scuola Normale Superiore, Piazza San Silvestro 12, 56127 Pisa, Italy; valentina.delorenzi@sns.it; 2 Islet Cell Laboratory, Department of Clinical and Experimental Medicine, University of Pisa, 56126 Pisa, Italy; marta.tesi91@gmail.com (M.T.); piero.marchetti@unipi.it (P.M.)

**Keywords:** pro-inflammatory cytokines, Airyscan, super resolution, β cells, fluorescence, endoplasmic reticulum, golgi apparatus

## Abstract

Pro-inflammatory cytokines play a role in the failure of β cells in type 1 and type 2 diabetes. While existing data from ‘omics’ experiments allow for some understanding of the molecular mechanisms behind cytokine-induced dysfunction in β cells, no report thus far has provided information on the direct imaging of the β cell landscape with nanoscale resolution following cytokine exposure. In this study, we use Airyscan-based optical super-resolution microscopy of Insulinoma 1E (INS-1E) cells to investigate the structural properties of two subcellular membranous compartments involved in the production, maturation and secretion of insulin-containing granules, the endoplasmic reticulum (ER) and the Golgi apparatus (GA). Our findings reveal that exposure of INS-1E cells to IL-1β and IFN-γ for 24 h leads to significant structural alterations of both compartments. In more detail, both the ER and the GA fragment and give rise to vesicle-like structures with markedly reduced characteristic area and perimeter and increased circularity with respect to the original structures. These findings complement the molecular data collected thus far on these compartments and their role in β cell dysfunction and lay the groundwork for future optical microscopy-based ex vivo and in vivo investigations.

## 1. Introduction

Pancreatic β cells play an indispensable role in maintaining glucose homeostasis in the body [1]. These specialized cells, located in the islets of Langerhans within the pancreas, are the exclusive producers of insulin, a hormone crucial for the regulation of blood glucose levels. In fact, insulin facilitates the uptake of glucose by tissues and reduces glucose production by the liver and other organs, thereby lowering blood glucose concentrations [1]. Insulin is produced by β cells in its precursor form, namely preproinsulin [2]. Preproinsulin is cleaved in the endoplasmic reticulum (ER) to form proinsulin [3,4], which is subsequently translocated to the Golgi apparatus (GA) where it is packaged into early secretory vesicles [5,6]. These immature vesicles undergo a series of maturation steps [7,8,9] and are compartmentalized in the cytoplasm until the mobilization/release step stimulated by exposure to glucose or other secretagogues [10]. Indeed, dysregulation of insulin production and/or secretion by β cells is a hallmark of both type 1 and type 2 diabetes (T1D and T2D), chronic conditions characterized by persistent hyperglycemia [11]. β cells devote most of their total protein biosynthetic capacity to insulin production [12,13,14] and can produce as much as 106 molecules of preproinsulin per minute following glucose stimulation [12]. Consequently, they possess a well-developed ER and are in turn highly susceptible to ER stress under conditions of increased insulin demand. Several studies report on the presence of ER stress in pancreatic islets from both diabetic mouse models and diabetic human subjects [15,16]. In addition, significant ER stress has been observed in immortalized insulin-producing cells and human pancreatic islets exposed to pro-inflammatory cytokines [17]. By contrast, the role and extent of GA stress in the pathophysiology of insulin secretion are still largely unknown [18]. Interestingly, a recent bioinformatic study on publicly available datasets from human T1D and T2D islet models has evidenced that GA-associated genes are dysregulated in diabetes [18]. Similarly, exposure to lipotoxicity and glucolipotoxicity has been shown to induce alterations in the expression of Golgi structural proteins, GA glycosylation enzymes and GA stress mediators in immortalized rat insulinoma cells [19]. Both the ER and the GA are highly dynamic structures that exhibit functional and morphological modifications in response to changes in cellular conditions such as developmental stage, intracellular signals or pathological conditions [20,21]. Thus far, the structural properties of the ER and the GA under diabetes-mimicking conditions have been evaluated almost exclusively by means of electron microscopy (EM). Several studies reported morphological alterations of the beta cell ER in diabetic mouse models [22,23,24,25,26] and T2D subjects [27,28]. On the other hand, only a few studies have focused on imaging the morphological properties of the GA under diabetes-mimicking conditions. Using EM, Alcaron and coworkers observed an expansion of the GA in a mouse model of obesity-linked T2D [26]. More recently, Boyer and coworkers revealed significant morphological abnormalities in the GA of diabetic mice lacking leptin receptors, including shortened and swollen cisternae, fewer cisternae per Golgi stack, and partial vesiculation of the GA [20].

In the present study, building on a recent report [29], we couple the molecular specificity of antibody-based recognition with super-resolution microscopy to study the structural alterations of the ER and the GA in INS-1E cells exposed to pro-inflammatory cytokines. The results show significant morphological alterations of both the ER and GA structures. In detail, after exposure to IL-1β and IFN-γ for 24 h, both organelles display vesicular fragmentation, with a marked reduction in the characteristic area and perimeter of the resulting structures and an increase in their circularity compared to control cells. In addition, under the same conditions, we occasionally observed organized smooth ER (OSER) structures in cells, in which the ER forms stacked membranes by integrating pre-existing branching ER [30,31]. Overall, the ER and GA alterations complement the structural modifications we recently observed in microtubules and mitochondria together with the reduced number of insulin granules under cytokine exposure [29], providing a comprehensive picture of the subcellular landscape of β cells in response to cytokine treatment.  

## 2. Results and Discussion

**Figure 1:**
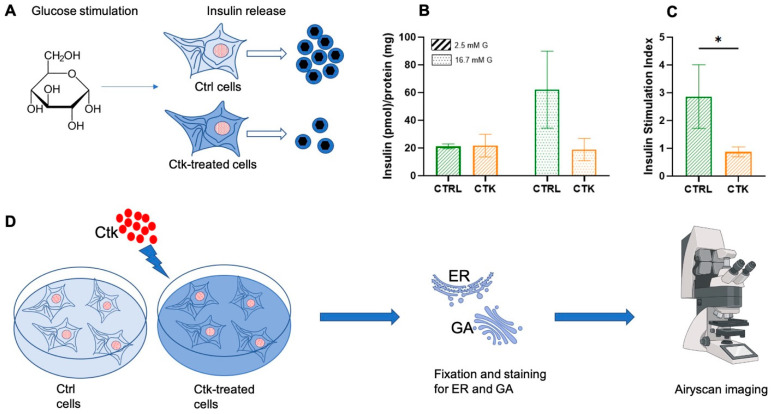
Schematic representation of the general workflow of our experiments. (**A**) After glucose stimulation, insulin secretion by INS-1E cells was evaluated. It was demonstrated that cytokine treatment impairs INS-1E insulin secretory response to glucose. (**B**) Effect of cytokine exposure on insulin release (corrected for protein content). (**C**) Effect of cytokine exposure on insulin stimulation index (ISI). Unpaired *t*-test, * *p* < 0.05. CTRL = control; CTK = cytokine; G = glucose. (**D**) INS-1E was plated and then incubated for 24 h in fresh complete medium or supplemented with cytokines (ctks). Then, the specimens were chemically fixed, stained with specific antibodies against the ER and GA and imaged using Airyscan microscopy (AM) (Jena, Germany).

The upregulation of pro-inflammatory cytokines is considered a hallmark in the pathophysiology of diabetes [32]. To study the effect of pro-inflammatory cytokines on β cells, we used INS-1E (Insulinoma 1E) cells. These cells exhibit many traits similar to primary β cells, such as glucose-sensing capability, making them a widely accepted model for β cells [33]. To verify the effectiveness of the cell system used, we evaluated the INS-1E insulin secretory response to glucose stimulation in normal conditions and after 24 h of cytokine treatment (Figure 1A).

At 2.5 mM glucose, no significant differences in insulin secretion were observed between control and cytokine-treated cells. However, when exposed to a high-glucose stimulus (16.7 mM), cytokine-treated cells displayed a markedly reduced insulin secretion capacity compared to control cells (Figure 1B). Consequently, the insulin stimulation index was lower in cytokine-treated cells compared to control samples (Figure 1C), in accordance with previous observations [34,35]. At this point, we conducted experiments to evaluate the possible alterations of the target cellular structures following cytokine treatment (Figure 1D). After treating the INS-1E cells with cytokines for 24 h, we fixed and stained them for the ER and GA to observe any structural changes. All the samples were imaged with an inverted Zeiss LSM 800 confocal microscope (Zeiss, Jena, Germany), equipped with an Airyscan detection unit to achieve super-resolution (see Section 3 for more details). To observe the ER, cells were immunostained with SERCA2 ATPase (Sarco-Endoplasmic Reticulum Calcium ATPase) monoclonal antibody, and imaged using Airyscan microscopy. Subsequently, morphometric analysis of the ER was conducted by comparing cytokine-treated cells with control cells.

**Figure 2:**
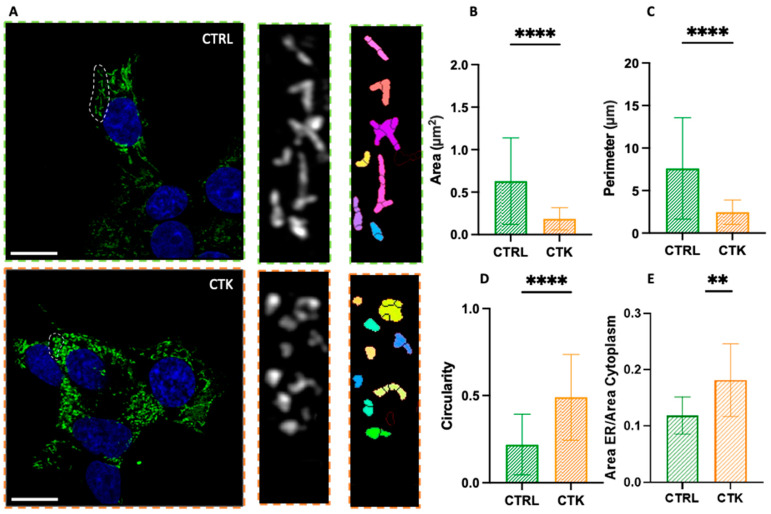
Airyscan imaging of the ER. (**A**) Confocal images of the endoplasmic reticulum network, with algorithmic segmentation executed via the MorphoLibJ plugin, observed in both control and cytokine-treated INS-1E cells. Analysis of endoplasmic reticulum vesicles’ structure (see exemplary regions highlighted by the dashed circles in both CTRL and CTK images) reveals a decrease in area and perimeter ((**B**) and (**C**), respectively), along with an increased circularity value (approaching 1) (**D**) in samples treated with cytokines. In this analysis, regions of the cells exhibiting visible vesicles were scrutinized (number of vesicles = 128 for control and 132 for treated samples; across n = 3 independent experiments). Statistical analysis was performed using a Mann–Whitney test (**** *p* < 0.0001). (**E**) The ratio of the ER area to the cytoplasm area is greater in cells treated with cytokines. Number of cells = 12 for control and 12 for treated samples across n = 3 independent experiments. Statistical analysis was performed using a parametric t test (** *p* < 0.01). Scale bar indicates 10 µm.

As shown in Figure 2A, in control cells, the ER has a structure composed of a set of membranous vesicles, which is more or less branched. On the contrary, cytokine-treated cells display an altered morphology characterized by extensive ER fragmentation, with the formation of more rounded and smaller structures, hereafter referred to as ‘vesicles’ or ‘vesicle-like structures’. This observation is quantitatively evident in the analysis of parameters such as the area, perimeter and circularity (Figure 2B–D) of the ER structures: the typical area changed from 0.63 ± 0.51 µm^2^ in control cells to 0.19 ± 0.13 µm^2^ in cytokine-treated cells (i.e., ~70% reduction); the perimeter changed from 7.60 ± 5.95 µm in control cells to 2.45 ± 1.43 µm in cytokine-treated cells (i.e., ~70% reduction); and the circularity changed from 0.22 ± 0.17 in control cells to 0.49 ± 0.24 in cytokine-treated cells (i.e., ~2-fold increase). The data thus show that this structure undergoes profound morphological changes upon exposure to pro-inflammatory cytokines and this might have consequences for insulin synthesis and then secretion. Additionally, in accordance with previous studies [25,26,27], we found an increase in the total area occupied by the ER in the cell (Figure 2E). Indeed, our immunofluorescence observations are in agreement with previous results obtained using electron microscopy [27], showing that ER density volume was significantly increased in T2D β cells [27]. Worthy of mention, in cytokine-exposed cells, we occasionally observed the presence of peculiar ER structures known as organized smooth ER (i.e., OSER) (Figure S1). The formation of OSER entails the integration of pre-existing branching ER into stacked structures [30,31]. The function of OSER structures in cells is not fully understood, but they seem to retain proteins longer than branching ER. This may be due to their fewer connections, which might cause protein segregation and compartmentalization within the ER [30]. To our knowledge, while these structures have been observed previously in other cells and under pathological conditions [30], this is the first documented occurrence in diabetes-mimicking conditions. Overall, these altered ER features could contribute to the diabetic β cell phenotype. The role of the ER in β cell pathophysiology is well documented. Islets exposed to metabolic stress and/or from T2D patients show altered ER morphology [27,36] and increased expression of genes involved in the ER stress response [37]. In the context of T1D, signs of ER stress have been found in the islets of type 1 diabetes patients [36,38] as well as in those from ND donors exposed to pro-inflammatory stimuli ex vivo [17,39].

**Figure 3:**
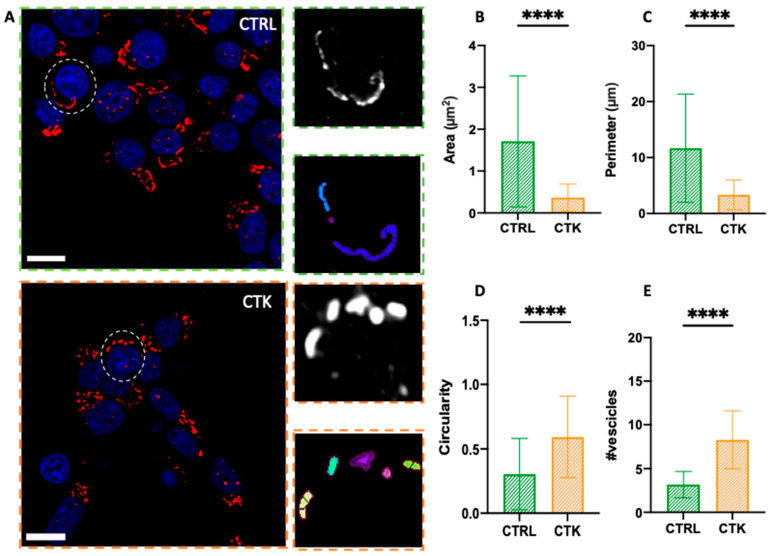
Airyscan imaging of the GA. (**A**) Golgi apparatus network confocal images and algorithm segmentation in control and cytokine-treated INS-1E cells. Area, perimeter and circularity of the single vesicles are shown in the tables. The structural analysis of GA vesicles (see exemplary regions highlighted by the dashed circles in both CTRL and CTK images) shows reduced area and perimeter ((**B**) and (**C**), respectively), high circulatory value (tending to 1) (**D**) and higher number of vesicles (**E**) in cytokine-treated samples. These results support the idea of vesicle fragmentation after cytokine treatment (number of cells = 45 for control and treated samples; n = 3 independent experiments). A Mann–Whitney test was performed (**** *p* < 0.0001). Cells were acquired by confocal microscope using 405 and 488 excitation light, with 63X/NA1.4 objective lens. Scale bar 10 µm.

To study GA morphology following 24 h of pro-inflammatory cytokine treatment, INS-1E cells were fixed, immunostained with a GM130 antibody, and imaged using Airyscan microscopy. Similarly to that observed for the ER, INS-1E cells exposed to cytokines exhibited extensive fragmentation of the GA, with the formation of vesicle-like structures (Figure 3A). Quantitatively, the area of the observed structures decreased from 1.71 ± 1.56 µm² in control cells to 0.36 ± 0.32 µm² in cytokine-treated cells (i.e., ~80% reduction) (Figure 3B); the perimeter decreased from 11.67 ± 9.68 µm in control cells to 3.33 ± 2.67 µm in cytokine-treated cells (i.e., ~70% reduction) (Figure 3C); and structure circularity increased from 0.30 ± 0.28 in control cells to 0.59 ± 0.32 in cytokine-treated cells (i.e., ~2-fold increase) (Figure 3D). Furthermore, the fragmentation process caused an increase in the average number of vesicles present in each cell, from N~3 in control cells to N~8 in cytokine-treated cells (Figure 3E), in accordance with previous ultrastructural analysis by Boyer and coworkers, performed using EM [40]. Considering the fundamental role of the GA in insulin maturation and thus β cell function, these alterations could contribute to diabetic β cell failure. Indeed, the expression of several GA-associated genes changes in both T1D and T2D islets [18] and specific mutations of some of those genes are also the cause of a few monogenic forms of diabetes [41].

## 3. Materials and Methods

Cell culture: INS-1E cells (kindly provided by Prof. C. Wollheim, University of Geneva, Medical Center) were maintained in culture at 37 °C, 5% CO2, in RPMI 1640 medium containing 11.1 mM D-glucose, supplemented with 10% heat-inactivated fetal bovine serum (FBS), 10 mM HEPES, 2 mM L-Glutamine, 100 U/mL penicillin–streptomycin, 1 mM sodium pyruvate and 50 μM tissue-culture-grade β mercaptoethanol (all purchased from Gibco, ThermoFisher). INS-1E cells were plated at 70% confluency on 18 mm coverglass and grown for 48 h. For each experiment carried out, a pair of samples was used: one control and one treated with cytokines (IL-1β 10 U/mL and IFN-γ 100 U/mL, diluted in 1 mL of complete medium RPMI) for 24 h. For control samples, cells were washed and replaced with fresh complete medium. Cells used in this study were negative for the presence of mycoplasma contamination.

Insulin secretion: Insulin release in response to glucose (2.5 and 16.7 mmol/L) was assessed as previously reported [42]. Briefly, cells were washed twice with Krebs’ buffer containing 2.5 mmol/L glucose and pre-incubated with the same buffer for 45 min at 37 °C. After another washing step, cells were incubated with Krebs’ buffer containing 2.5 mmol/L or 16.7 mmol/L glucose for 30 min at 37 °C. Insulin was quantified in cell supernatant by a High Range Rat Insulin ELISA kit (Mercodia AB, Uppsala, Sweden) and insulin release was normalized by protein content. Proteins were extracted with RIPA buffer (Thermo Fisher Scientific, Waltham, MA, USA) and quantified by a Pierce™ BCA Protein Assay Kit (Thermo Fisher Scientific). The insulin stimulation index (ISI) was calculated as the ratio of insulin release at 16.7 mmol/L glucose over the release at 2.5 mmol/L glucose.

Immunostaining: Control and cytokine-treated cells were fixed with 4% paraformaldehyde (PFA) in PBS for 30 min at RT and washed 3 times with PBS, 5 min each. After fixation, cells were permeabilized through 3 washes of 5 min each at RT with PBS + 0.1% Triton X-100 (PBST). Cells were then washed 3 times with PBS and blocked with 2% bovine serum albumin (BSA) for 30–45 min at RT. The samples were incubated with the following primary antibodies in a humidified chamber: anti-GM120 antibody for GA (ARG57583, Arigo Biolaboratories, Taiwan) diluted 1:100 in PBS + 0.1% Tween overnight at 4 °C, and SERCA2 ATPase Monoclonal Antibody for ER (MA3-919, Thermo Fisher Scientific, Waltham, MA, USA) diluted 1:100 in PBS + 0.1% Tween overnight at 37 °C. Then, after 1 wash with PBS + 0.1% Tween and 2 washes with PBS of 5 min each at RT, the specimens were stained for 1 h at RT with secondary antibodies: anti-rabbit Alexa Fluor 488 ((IS-20015-1, Immunological Sciences, Rome, Italy) for GA and anti-mouse Alexa Fluor 488 (IS-20014-1, Immunological Sciences, Rome, Italy) for ER, diluted 1:100 in PBS + 0.1% tween. The stained samples were then washed 2 times with PBS, 10 min each, and then incubated with 1 μg/mL DAPI in PBS for 10 min. Specimens were mounted in Mowiol before imaging.

Fluorescence microscopy: Microscopic observation and photography were performed using an inverted Zeiss LSM 800 confocal microscope (Jena, Germany), equipped with an Airyscan detection unit. The acquisition was performed by illuminating the sample with a 405 and 488 laser using a 63X/NA 1.4 oil-immersion objective. DAPI and Alexa Fluor 488 fluorescence were collected between 410 and 510 nm and between 510 and 590 nm, respectively, with GaAsP detectors. The pinhole aperture was set at 1 Airy (53 μm). Detector gain and pixel dwell times were adjusted for each dataset, keeping them at their lowest values in order to avoid saturation and bleaching effects. Imaging was performed using Airyscan microscopy (AM) which allows for simultaneous improvement in resolution and signal-to-noise without increasing the excitation power and acquisition time. AM offers fast and sensitive super-resolution confocal imaging, which improves lateral resolution to 120 nm. Thus, it is filling the gap between classical confocal laser scanning microscopy and super-resolution structured-illumination microscopy (SIM). Instead of employing a single photomultiplier tube (PMT) commonly found in traditional confocal microscopes, AM utilizes a specialized configuration of 32-channel gallium arsenide phosphide (GaAsP) detectors arranged in a hexagonal array. Each detection element within the GaAsP detector operates like a small pinhole, measuring 0.2 Airy units (AU) in size. The overall collection efficiency amounts to approximately 1.25 AU.

Data analysis: The morphology of the GA and ER was analyzed by selecting whole cells and specific regions within the cells, respectively. These selected images underwent processing using Fiji 2.9.0 to enhance the signal-to-noise ratio (SNR) through background subtraction and Gaussian filtering. Subsequently, the MorphoLibJ plugin (version 1.6.2) was employed for analysis by inputting “Object Image” and setting appropriate tolerance values for each Region of Interest (ROI). This process generated a binary image, which was then subjected to analysis using the MorphoLibJ “Analyze” function to extract information on the organelles’ area, perimeter and circularity. To determine the ratio of the endoplasmic reticulum (ER) area to cytoplasmic area, cells were selected by drawing a Region of Interest (ROI) around their perimeter in Fiji 2.9.0, and the total cell area was measured using “Analyze > Measure.” The selected cells were processed to enhance the signal-to-noise ratio through background subtraction. A new ROI was then drawn around the nucleus to measure its area, which was subtracted from the total cell area to obtain the cytoplasmic area. To identify ER pixels within the ROI, the “Image > Adjust > Threshold” function was used, and after applying the threshold, a selection was created with “Edit > Selection > Create Selection”. To retain only the ER within the original ROI, “Edit > Clear Outside” was employed. The ER area within the original ROI was measured using “Analyze > Measure”, and the ratio of ER area to cytoplasmic area was calculated.

## 4. Conclusions

To conclude, the cytokine-induced ER and GA alterations highlighted here complement the structural modifications of microtubules (i.e., extensive fragmentation) and mitochondria (i.e., increased circularity) and the altered number of insulin granules (i.e., reduced) recently observed by some researchers under the same experimental conditions and by means of a similar optical microscopy approach [29]. Taken together, these observations provide a comprehensive picture of the subcellular landscape of β cells in response to treatment with pro-inflammatory cytokines.

## Data Availability

Data will be made available upon request to the corresponding author, Francesco Cardarelli (francesco.cardarelli@sns.it).

## Supplementary Materials

The following supporting information can be downloaded at: https://www.mdpi.com/article/10.3390/ijms251910391/s1.
