# Supplementary material for: Optical Nanoscopy of Cytokine-Induced Structural Alterations of the Endoplasmic Reticulum and Golgi Apparatus in Insulin-Secreting Cells
Source: Int J Mol Sci. 2024 Sep 27;25(19):10391. doi: 10.3390/ijms251910391 (PMC11476361; doi:10.3390/ijms251910391)
Supplement: Supplementary file 1 [file ijms-25-10391-s001.zip › ijms-3175365-supplementary.pdf]

Supplementary Figure

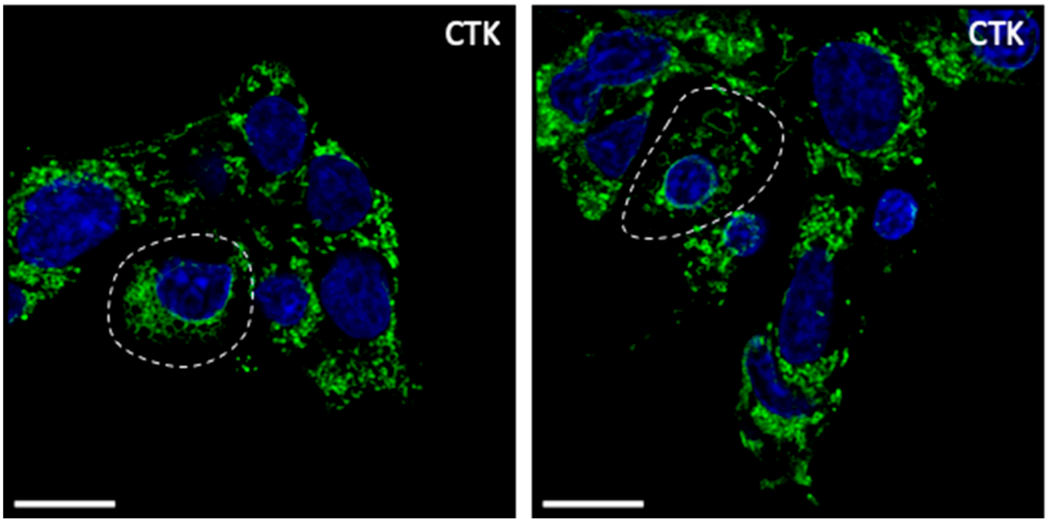

Figure S1. Airyscan imaging of OSER structures. Representative images of the OSER structures of ER in INS1-E cells treated with cytokines (Il-1β and IFN-γ) for 24h. Scale bar indicates 10 μm.
